# Endometrial Receptivity During the Preimplantation Period: A Narrative Review

**DOI:** 10.7759/cureus.37753

**Published:** 2023-04-18

**Authors:** Kshitij Bajpai, Neema Acharya, Roshan Prasad, Mayur B Wanjari

**Affiliations:** 1 Medicine, Jawaharlal Nehru Medical College, Datta Meghe Institute of Higher Education and Research, Wardha, IND; 2 Obstetrics and Gynecology, Jawaharlal Nehru Medical College, Datta Meghe Institute of Higher Education and Research, Wardha, IND; 3 Medicine and Surgery, Jawaharlal Nehru Medical College, Datta Meghe Institute of Higher Education and Research, Wardha, IND; 4 Research and Development, Jawaharlal Nehru Medical College, Datta Meghe Institute of Higher Education and Research, Wardha, IND

**Keywords:** exosomes, mesenchymal stem cells, proteomics, transcriptomics, biomarkers, infertility, endometrial receptivity

## Abstract

Endometrial receptivity is a complex and critical process fundamental to achieving a successful pregnancy. While researchers have made significant strides in understanding the underlying mechanisms governing endometrial receptivity, effective diagnostic and therapeutic strategies remain scarce. This review article aims to elucidate the various factors that contribute to endometrial receptivity, including the hormonal regulation and molecular mechanisms that govern this process, as well as potential biomarkers for assessing endometrial receptivity. One of the major challenges in identifying reliable biomarkers for endometrial receptivity is the intricate nature of the process itself. Nonetheless, recent advances in transcriptomic and proteomic technologies have identified several candidate biomarkers that could potentially enhance our ability to predict endometrial receptivity. Furthermore, emerging technologies such as single-cell RNA sequencing and mass spectrometry-based proteomics hold great promise for providing novel insights into the molecular mechanisms underlying endometrial receptivity. Despite the lack of reliable biomarkers, various therapeutic strategies have been proposed to improve endometrial receptivity. One promising approach involves the transplantation of mesenchymal stem cells (MSCs), which have been shown to increase endometrial thickness and receptivity in both animal models and clinical trials. Growth factors, cytokines, and exosomes derived from MSCs and other cell types may also have therapeutic potential for addressing endometrial dysfunction.

## Introduction and background

Implantation of a fertilized embryo into the endometrium constitutes a critical milestone in establishing a successful pregnancy. The concept of endometrial receptivity alludes to the specific period during the menstrual cycle when the endometrium can receive and accommodate implantation by a developing embryo. Endometrial receptivity is a dynamic and tightly regulated process orchestrated by a complex interplay of molecular, cellular, and hormonal mechanisms. The comprehension of factors that influence endometrial receptivity is of utmost significance in advancing the success rates of assisted reproductive technologies and the development of novel infertility therapies [1].

The menstrual cycle is a multifaceted physiological phenomenon controlled by the hypothalamus-pituitary-ovarian (HPO) axis, which regulates the maturation and release of oocytes from the ovary, endometrial thickening, and endometrial lining shedding in the absence of implantation. The endometrium undergoes cyclic alterations in response to estrogen and progesterone levels throughout the menstrual cycle. The proliferative phase is characterized by the growth and thickening of the endometrium, driven by estrogen. In contrast, the secretory phase is marked by the differentiation of the endometrial glands and stroma, preparing the endometrium for implantation under the influence of progesterone [2].

Endometrial receptivity is a phenomenon that occurs during the secretory phase of the menstrual cycle when the endometrium becomes receptive to the implantation of a developing embryo. This process is highly regulated and occurs within a narrow time frame, typically between days 19 and 21 of a 28-day menstrual cycle [3]. During this period, a transient shift in gene expression and molecular signaling within the endometrium prepares it for the successful implantation of a fertilized embryo. The establishment of endometrial receptivity is crucial for successful implantation, and any disruption in this process can result in implantation failure or early pregnancy loss [4].

The molecular mechanisms underlying endometrial receptivity involve intricate interactions between the embryo and the endometrium. The embryo secretes several signaling molecules, including human chorionic gonadotropin (HCG), cytokines, and growth factors, stimulating the endometrium to become receptive to implantation [5]. The endometrium, in turn, expresses a set of genes and proteins that facilitate embryo implantation and early development. These include adhesion molecules, cytokines, chemokines, and growth factors [6]. A failure in any of these signaling pathways can lead to implantation failure, early pregnancy loss, or other pregnancy complications.

Endometrial receptivity biomarkers refer to a collection of genes and proteins differentially expressed during the endometrial receptivity window. These biomarkers can evaluate endometrial receptivity and anticipate the ideal timing for embryo transfer in assisted reproductive techniques (ARTs). Several biomarkers have been identified, such as leukemia inhibitory factor (LIF), Homeobox A10 (HOXA10), integrin beta 3 (ITGβ3), and fibroblast growth factor 18 (FGF18). The expression of these biomarkers is governed by hormonal and molecular signaling pathways and is influenced by various physiological and pathological conditions [7].

The assessment of endometrial receptivity is crucial for improving the success rates of ARTs. Various methods are available to evaluate endometrial receptivity, including histological examination, imaging techniques, and molecular and genomic approaches. Histological evaluation entails analyzing the endometrial tissue under a microscope and evaluating its morphology and cellular composition [8]. Imaging techniques, such as ultrasound and magnetic resonance imaging (MRI), can visualize endometrial thickness, vascularity, and other features that indicate endometrial receptivity [9]. Molecular and genomic approaches involve assessing the expression of endometrial receptivity biomarkers using techniques such as microarray analysis, ribonucleic acid (RNA) sequencing, and quantitative polymerase chain reaction (qPCR) [10]. These methods have enabled researchers and clinicians to identify and quantify the expression of biomarkers that predict endometrial receptivity, allowing for better timing of embryo transfer and improved pregnancy outcomes.

Endometrial receptivity can be affected by a range of physiological and pathological conditions. As observed in polycystic ovary syndrome (PCOS), hormonal imbalances can interfere with endometrial receptivity and contribute to failed implantation [11]. Endometrial pathologies, including endometriosis and polyps, can also impact endometrial receptivity and increase the risk of pregnancy complications [12]. Additionally, systemic conditions, such as obesity and diabetes, may adversely affect endometrial receptivity [13].

In ARTs, assessing endometrial receptivity carries significant clinical implications. Biomarkers of endometrial receptivity can aid clinicians in determining the optimal timing for embryo transfer, which reduces the risk of implantation failure and enhances the success rates of in vitro fertilization (IVF) and other ARTs [14]. Furthermore, a more profound understanding of the molecular mechanisms governing endometrial receptivity could lead to the development of novel therapeutic approaches for infertility. This narrative review article aims to summarize and critically evaluate the current literature on endometrial receptivity during the preimplantation period. Specifically, this article aims to provide an overview of the hormonal and molecular mechanisms that regulate endometrial receptivity, the various factors that can impact this process, and the potential biomarkers that can be used to assess endometrial receptivity. Additionally, this review aims to highlight the importance of understanding endometrial receptivity in achieving successful pregnancy outcomes and to identify areas for future research in this field.

## Review

Methodology

This study was conducted through a comprehensive scientific literature search using various electronic databases such as PubMed, Embase, and Cochrane Library. The search was carried out by employing a combination of relevant keywords and medical subject heading (MeSH) terms, which included "endometrial receptivity," "implantation," "menstrual cycle," "hormones," "molecular mechanisms," "biomarkers," "assisted reproductive techniques," and "infertility."

The inclusion criteria for the articles consisted of original research articles, review articles, and meta-analyses that pertained to endometrial receptivity, implantation, and infertility. Moreover, articles that focused on the menstrual cycle, hormones, molecular mechanisms, biomarkers, and ARTs and were published in peer-reviewed journals were also included.

On the other hand, the exclusion criteria for the articles included articles published in non-peer-reviewed journals and articles published in languages other than English. These criteria were applied to ensure that only relevant and high-quality literature was included in this study.

Factors affecting endometrial receptivity

The establishment of endometrial receptivity is critically dependent on hormonal regulation. The menstrual cycle, governed by the HPO axis, orchestrates cyclic changes in the endometrium in response to fluctuations in estrogen and progesterone levels [6]. Progesterone is essential during the secretory phase of the menstrual cycle to establish and maintain endometrial receptivity. Disruptions in hormonal balance, as observed in PCOS, can interfere with endometrial receptivity and impede implantation [15,16].

Signaling pathways play a critical role in the success or failure of pregnancy. In normal pregnancies, the embryo releases various signaling molecules that interact with the endometrium, leading to implantation and successful pregnancy. However, in certain cases, abnormalities in these signaling pathways can result in pregnancy failure or other complications [15,16]. For instance, the dysregulation of cytokines and growth factors crucial for successful embryo implantation can lead to implantation failure or early pregnancy loss. Additionally, abnormalities in the expression of adhesion molecules in the endometrium can hinder the embryo's attachment to the endometrial lining, leading to implantation failure. Similarly, altered expression of chemokines can lead to abnormal immune responses, resulting in the maternal immune system rejecting the embryo. These mechanisms can also contribute to pregnancy complications such as preeclampsia and preterm labor. The pathophysiology of pregnancy failure associated with dysregulated signaling pathways is multifactorial and complex. It involves various factors, such as immune dysfunction, endocrine imbalances, genetic abnormalities, and environmental factors. Therefore, further research is needed to fully understand the mechanisms involved in pregnancy failure and develop effective interventions to prevent it [5,17].

Endometrial receptivity biomarkers are HOXA10, LIF, and ESR1, as well as integrins, mucins, and selectin-specific genes and proteins that are differentially expressed during the window of endometrial receptivity. These biomarkers can assess endometrial receptivity and predict the optimal timing for embryo transfer in ARTs. The expression of these biomarkers is regulated by both hormonal and molecular signaling pathways and is influenced by various physiological and pathological conditions [7,18].

Methods for assessing endometrial receptivity

The accurate assessment of endometrial receptivity is crucial for successful implantation and pregnancy in ARTs such as IVF [19]. Therefore, evaluating endometrial receptivity using modern noninvasive methods that provide adequate information on the molecular and genomic factors involved is essential. This study examines various methods for assessing endometrial receptivity, including histological evaluation, imaging techniques, and molecular and genomic approaches [20].

Histological evaluation has been the gold standard for assessing endometrial receptivity for many years. This method involves an endometrium biopsy, which is then examined under a microscope for morphological changes [19,20]. The morphological changes observed in the endometrium indicate its receptivity to an embryo. However, this method is invasive, uncomfortable, and has a risk of complications. Additionally, it may not provide the necessary information on molecular and genomic changes in the endometrium required for successful implantation [21].

Imaging techniques, namely, ultrasound and MRI, have been employed to evaluate endometrial receptivity [19]. Ultrasound is an economical and noninvasive technique that gauges endometrial thickness, pattern, and blood flow. Endometrial thickness is a crucial parameter for evaluating endometrial receptivity. However, ultrasound has limited potential in providing molecular and genomic details of the endometrium [22].

MRI is a noninvasive imaging technique that offers high-resolution images of the endometrium. It can offer information on endometrial thickness, pattern, and vascularity, akin to ultrasound. However, it can also provide molecular and genomic insights into the endometrium, which are fundamental for evaluating endometrial receptivity. Additionally, MRI can be employed to assess the endometrium's response to hormonal stimulation, essential for evaluating endometrial receptivity [1].

Molecular and genomic approaches have emerged as promising methods for evaluating endometrial receptivity. These methods involve the analysis of specific genes, proteins, and other molecular factors involved in endometrial receptivity, such as cytokines, growth factors, adhesion molecules, and extracellular matrix proteins [15]. Molecular and genomic approaches utilize advanced techniques, such as PCR, microarray analysis, and next-generation sequencing (NGS), which allow for the assessment of gene expression patterns and identification of biomarkers that can be used to evaluate endometrial receptivity [19].

The gold standard method for evaluating endometrial receptivity is histological evaluation. Endometrial biopsies are taken for this purpose during the window of implantation (WOI), which typically occurs between days 19 and 21 of a 28-day menstrual cycle [23]. Histological evaluation involves the analysis of endometrial tissue to assess morphological changes during the WOI, including glandular and stromal development, vascularization, and immune cell infiltration. Although an invasive procedure that can be associated with discomfort and bleeding, endometrial biopsy for histological evaluation is still considered the most reliable method for assessing endometrial receptivity [24].

Noninvasive imaging techniques, such as ultrasound and MRI, have been demonstrated as effective methods for evaluating endometrial receptivity. Ultrasound can be used to assess endometrial thickness and pattern and the presence of uterine and ovarian abnormalities [25]. Transvaginal ultrasound is particularly advantageous in assessing endometrial receptivity because it provides information on endometrial blood flow and fluid collection. While MRI can also evaluate endometrial thickness and pattern and the presence of uterine and ovarian abnormalities, it is generally more costly and less readily available than ultrasound [24].

Molecular and genomic approaches are emerging as promising methods for assessing endometrial receptivity. Molecular markers, such as integrins and cytokines, can provide insight into the molecular mechanisms involved in endometrial receptivity [24,26]. Genomic approaches, including gene expression profiling and microRNA analysis, offer comprehensive information on the molecular pathways that regulate endometrial receptivity. These approaches are noninvasive and provide more comprehensive information than histological evaluation or imaging techniques. However, they are still in the early stages of development and require further validation before they can be widely used in clinical practice [27,28].

Impact of endometrial receptivity on fertility outcomes

The success of embryo implantation and subsequent pregnancy depends on endometrial receptivity [29]. The complex interplay of molecular events between the developing embryo and the endometrium is integral to embryo implantation. Optimal endometrial conditions are necessary for successful embryo implantation, and any abnormalities in endometrial receptivity can result in implantation failure and infertility [30].

Numerous studies have extensively explored the role of endometrial receptivity in embryo implantation and identified several factors that affect the endometrial receptivity window. Hormonal regulation of endometrial receptivity is a crucial factor that plays a critical role in establishing a receptive endometrial environment [31]. Estrogen and progesterone levels in the endometrium are necessary for preparing the endometrium for embryo implantation. Inadequate levels of these hormones have been reported to cause implantation failure and infertility in several studies [32].

The establishment of a receptive endometrial environment is dependent on molecular mechanisms that are involved in endometrial receptivity. These mechanisms govern gene expression, cytokine signaling, and extracellular matrix remodeling, and their dysregulation can result in impaired endometrial receptivity and implantation failure [33].

To determine the optimal timing for embryo transfer, it is crucial to assess biomarkers of endometrial receptivity [7]. Various biomarkers that can be used to predict endometrial receptivity have been identified, such as the measurement of endometrial thickness, uterine artery blood flow, and the expression of certain genes and proteins. These biomarkers can improve the accuracy of embryo transfer timing and increase the success rates of ARTs [14].

The clinical implications of assessing endometrial receptivity in ART have been extensively researched, and multiple studies have reported a significant enhancement in pregnancy rates through endometrial receptivity assessment [7,33]. Utilizing biomarkers to predict endometrial receptivity has been demonstrated to enhance embryo transfer success rates and decrease the incidence of implantation failure [14].

Future directions

Endometrial receptivity is a multifaceted and intricate event involving the synchronization of various molecular and cellular activities. Its dysfunction is a notable factor contributing to infertility. Despite considerable progress in comprehending the underlying mechanisms involved in endometrial receptivity, there remains a considerable gap in developing effective diagnostic and therapeutic approaches. This section explores the up-and-coming technologies and potential therapeutic interventions for improving endometrial receptivity.

The principal challenge in evaluating endometrial receptivity lies in the inadequacy of dependable biomarkers. Despite extensive research, no single biomarker has been established to be adequately sensitive or specific to predict endometrial receptivity precisely. However, recent advancements in transcriptomic and proteomic technologies have identified numerous prospective biomarkers that may prove useful in predicting endometrial receptivity. For instance, a range of genes and proteins that are differentially expressed in the endometrium during the implantation window have been recognized in recent studies, and they have the potential to serve as biomarkers for assessing endometrial receptivity [34,35]. Other emerging technologies, such as single-cell RNA sequencing and mass spectrometry-based proteomics, may provide novel insights into the molecular mechanisms that govern endometrial receptivity [36,37].

In addition to enhancing our comprehension of the molecular mechanisms that underlie endometrial receptivity, there is the possibility that emerging technologies may provide novel opportunities for the development of therapeutic approaches to improve endometrial receptivity. One such strategy that has been investigated in recent studies is using mesenchymal stem cells (MSCs) to treat endometrial dysfunction [38]. MSCs possess immunomodulatory properties and are capable of differentiating into endometrial cells. Evidence indicates that the transplantation of MSCs can enhance endometrial thickness and receptivity in both animal models and clinical trials [39,40]. Additional therapeutic interventions for improving endometrial receptivity involve growth factors and cytokines, which have a significant role in regulating endometrial function and implantation [41].

Furthermore, research in recent times has explored the potential use of exosomes for treating endometrial dysfunction. Exosomes are small membrane-bound vesicles secreted by various cell types, containing a wide range of bioactive molecules such as proteins, lipids, and nucleic acids. Exosomes play critical roles in intercellular communication and cellular function regulation. Recent findings suggest that exosomes derived from MSCs and other cell types may possess therapeutic potential for treating endometrial dysfunction [42,43].

## Conclusions

In conclusion, the establishment of endometrial receptivity is a crucial stage in achieving a successful pregnancy, involving a complex interplay of hormonal, molecular, and cellular mechanisms. The molecular mechanisms underlying endometrial receptivity entail intricate interactions between the embryo and the endometrium, which entail the secretion of numerous signaling molecules by the embryo, and the expression of a set of genes and proteins by the endometrium. This process is tightly regulated and occurs during a brief period in the secretory phase of the menstrual cycle. Disruptions in these signaling pathways can result in implantation failure, early pregnancy loss, or other pregnancy complications. Consequently, comprehending the factors that regulate endometrial receptivity is critical to enhance the success rates of ARTs and develop new therapies for infertility. Endometrial receptivity is influenced by several factors, including hormonal regulation, molecular mechanisms, and endometrial receptivity biomarkers. Hormonal imbalances, such as those observed in PCOS, can interfere with the process of endometrial receptivity and result in implantation failure. Endometrial pathologies, such as endometriosis and polyps, can also impact endometrial receptivity and lead to pregnancy complications. Furthermore, systemic conditions, including obesity and diabetes, can negatively impact endometrial receptivity. Therefore, a comprehensive understanding of the mechanisms that control endometrial receptivity is necessary to optimize the chances of successful implantation and pregnancy.

## References

[1] Koot YE, Teklenburg G, Salker MS, Brosens JJ, Macklon NS (2012). Molecular aspects of implantation failure. Biochim Biophys Acta.

[2] Lessey BA (2000). Endometrial receptivity and the window of implantation. Baillieres Best Pract Res Clin Obstet Gynaecol.

[3] Noyes RW, Hertig AT, Rock J (1975). Dating the endometrial biopsy. Am J Obstet Gynecol.

[4] Brosens JJ, Gellersen B (2006). Death or survival--progesterone-dependent cell fate decisions in the human endometrial stroma. J Mol Endocrinol.

[5] Sherwin JR, Sharkey AM, Cameo P, Mavrogianis PM, Catalano RD, Edassery S, Fazleabas AT (2007). Identification of novel genes regulated by chorionic gonadotropin in baboon endometrium during the window of implantation. Endocrinology.

[6] Kao LC, Tulac S, Lobo S (2002). Global gene profiling in human endometrium during the window of implantation. Endocrinology.

[7] Miravet-Valenciano JA, Rincon-Bertolin A, Vilella F, Simon C (2015). Understanding and improving endometrial receptivity. Curr Opin Obstet Gynecol.

[8] Timmermans A, Opmeer BC, Khan KS (2010). Endometrial thickness measurement for detecting endometrial cancer in women with postmenopausal bleeding: a systematic review and meta-analysis. Obstet Gynecol.

[9] Järvelä IY, Sladkevicius P, Kelly S, Ojha K, Campbell S, Nargund G (2005). Evaluation of endometrial receptivity during in-vitro fertilization using three-dimensional power Doppler ultrasound. Ultrasound Obstet Gynecol.

[10] von Grothusen C, Lalitkumar S, Boggavarapu NR, Gemzell-Danielsson K, Lalitkumar PG (2014). Recent advances in understanding endometrial receptivity: molecular basis and clinical applications. Am J Reprod Immunol.

[11] Paulson RJ (2019). Introduction: Endometrial receptivity: evaluation, induction and inhibition. Fertil Steril.

[12] Lessey BA (2011). Assessment of endometrial receptivity. Fertil Steril.

[13] Leroy JL, Vanholder T, Mateusen B (2005). Non-esterified fatty acids in follicular fluid of dairy cows and their effect on developmental capacity of bovine oocytes in vitro. Reproduction.

[14] Haouzi D, Assou S, Dechanet C, Anahory T, Dechaud H, De Vos J, Hamamah S (2010). Controlled ovarian hyperstimulation for in vitro fertilization alters endometrial receptivity in humans: protocol effects. Biol Reprod.

[15] Lessey BA (1998). Endometrial integrins and the establishment of uterine receptivity. Hum Reprod.

[16] Psychoyos A (1973). Hormonal control of ovoimplantation. Vitam Horm.

[17] Critchley HO, Brenner RM, Henderson TA (2001). Estrogen receptor beta, but not estrogen receptor alpha, is present in the vascular endothelium of the human and nonhuman primate endometrium. J Clin Endocrinol Metab.

[18] Ruiz-Alonso M, Blesa D, Díaz-Gimeno P (2013). The endometrial receptivity array for diagnosis and personalized embryo transfer as a treatment for patients with repeated implantation failure. Fertil Steril.

[19] Haouzi D, Assou S, Mahmoud K (2009). Gene expression profile of human endometrial receptivity: comparison between natural and stimulated cycles for the same patients. Hum Reprod.

[20] Nardo LG, Gelbaya TA, Wilkinson H, Roberts SA, Yates A, Pemberton P, Laing I (2009). Circulating basal anti-Müllerian hormone levels as predictor of ovarian response in women undergoing ovarian stimulation for in vitro fertilization. Fertil Steril.

[21] Seli E, Sakkas D, Scott R, Kwok SC, Rosendahl SM, Burns DH (2007). Noninvasive metabolomic profiling of embryo culture media using Raman and near-infrared spectroscopy correlates with reproductive potential of embryos in women undergoing in vitro fertilization. Fertil Steril.

[22] Lessey BA, Young SL (2019). What exactly is endometrial receptivity?. Fertil Steril.

[23] Matthews ML (2015). Abnormal uterine bleeding in reproductive-aged women. Obstet Gynecol Clin North Am.

[24] Yao Y, Lv W, Xie X, Cheng X (2019). The value of hysteroscopy and transvaginal ultrasonography in the diagnosis of endometrial hyperplasia: a systematic review and meta-analysis. Transl Cancer Res.

[25] Tong R, Zhou Y, He Q, Zhuang Y, Zhou W, Xia F (2020). Analysis of the guidance value of 3D ultrasound in evaluating endometrial receptivity for frozen-thawed embryo transfer in patients with repeated implantation failure. Ann Transl Med.

[26] Lopes IM, Maganhin CC, Oliveira-Filho RM (2014). Histomorphometric analysis and markers of endometrial receptivity embryonic implantation in women with polycystic ovary syndrome during the treatment with progesterone. Reprod Sci.

[27] Horcajadas JA, Pellicer A, Simón C (2007). Wide genomic analysis of human endometrial receptivity: new times, new opportunities. Hum Reprod Update.

[28] Katzorke N, Vilella F, Ruiz M, Krüssel JS, Simón C (2016). Diagnosis of endometrial-factor infertility: current approaches and new avenues for research. Geburtshilfe Frauenheilkd.

[29] Lessey BA (2000). The role of the endometrium during embryo implantation. Hum Reprod.

[30] Singh M, Chaudhry P, Asselin E (2011). Bridging endometrial receptivity and implantation: network of hormones, cytokines, and growth factors. J Endocrinol.

[31] Brosens JJ, Pijnenborg R, Brosens IA (2002). The myometrial junctional zone spiral arteries in normal and abnormal pregnancies: a review of the literature. Am J Obstet Gynecol.

[32] Evans J, Salamonsen LA (2013). Too much of a good thing? Experimental evidence suggests prolonged exposure to hCG is detrimental to endometrial receptivity. Hum Reprod.

[33] Horcajadas JA, Mínguez P, Dopazo J (2008). Controlled ovarian stimulation induces a functional genomic delay of the endometrium with potential clinical implications. J Clin Endocrinol Metab.

[34] Hayashi KG, Hosoe M, Kizaki K, Fujii S, Kanahara H, Takahashi T, Sakumoto R (2017). Differential gene expression profiling of endometrium during the mid-luteal phase of the estrous cycle between a repeat breeder (RB) and non-RB cows. Reprod Biol Endocrinol.

[35] Zhang WB, Li Q, Liu H (2021). Transcriptomic analysis of endometrial receptivity for a genomic diagnostics model of Chinese women. Fertil Steril.

[36] Lai ZZ, Wang Y, Zhou WJ (2022). Single-cell transcriptome profiling of the human endometrium of patients with recurrent implantation failure. Theranostics.

[37] Segura-Benítez M, Carbajo-García MC, Corachán A, Faus A, Pellicer A, Ferrero H (2022). Proteomic analysis of extracellular vesicles secreted by primary human epithelial endometrial cells reveals key proteins related to embryo implantation. Reprod Biol Endocrinol.

[38] Lu X, Cui J, Cui L, Luo Q, Cao Q, Yuan W, Zhang H (2019). The effects of human umbilical cord-derived mesenchymal stem cell transplantation on endometrial receptivity are associated with Th1/Th2 balance change and uNK cell expression of uterine in autoimmune premature ovarian failure mice. Stem Cell Res Ther.

[39] Tan J, Li P, Wang Q (2016). Autologous menstrual blood-derived stromal cells transplantation for severe Asherman's syndrome. Hum Reprod.

[40] Tersoglio AE, Tersoglio S, Salatino DR, Castro M, Gonzalez A, Hinojosa M, Castellano O (2020). Regenerative therapy by endometrial mesenchymal stem cells in thin endometrium with repeated implantation failure. A novel strategy. JBRA Assist Reprod.

[41] Ozturk S, Demir R (2010). Particular functions of estrogen and progesterone in establishment of uterine receptivity and embryo implantation. Histol Histopathol.

[42] Gu X, Li Y, Chen K (2020). Exosomes derived from umbilical cord mesenchymal stem cells alleviate viral myocarditis through activating AMPK/mTOR-mediated autophagy flux pathway. J Cell Mol Med.

[43] Qiu G, Zheng G, Ge M, Wang J, Huang R, Shu Q, Xu J (2018). Mesenchymal stem cell-derived extracellular vesicles affect disease outcomes via transfer of microRNAs. Stem Cell Res Ther.

